# “Smart” Matrix Microneedle Patch Made of Self‐Crosslinkable and Multifunctional Polymers for Delivering Insulin On‐Demand

**DOI:** 10.1002/advs.202303665

**Published:** 2023-09-18

**Authors:** Jackie Fule Liu, Amin GhavamiNejad, Brian Lu, Sako Mirzaie, Melisa Samarikhalaj, Adria Giacca, Xiao Yu Wu

**Affiliations:** ^1^ Advanced Pharmaceutics and Drug Delivery Laboratory Leslie L. Dan Faculty of Pharmacy University of Toronto Toronto M5S 3M2 Canada; ^2^ Department of Physiology Faculty of Medicine University of Toronto Toronto M5S 1A8 Canada

**Keywords:** diabetes treatment, glucose‐responsive hydrogel, matrix microneedle patch, molecular dynamics modeling, self‐crosslinking

## Abstract

A transdermal patch that delivers insulin at high glucose concentrations can offer tremendous advantages to ease the concern of safety and improve the quality of life for people with diabetes. Herein, a novel self‐crosslinkable and glucose‐responsive polymer‐based microneedle patch (MN) is designed to deliver insulin at hyperglycemia. The microneedle patch is made of hyaluronic acid polymers functionalized with dopamine and 4‐amino‐3‐fluorophenylboronic acid (AFBA) that can be quickly crosslinked upon mixing of the polymer solutions in the absence of any chemicalcrosslinking agents or organic solvents. The catechol groups in the dopamine (DA) units form covalent crosslinkages among themselves by auto‐oxidation and dynamic crosslink with phenylboronic acid (PBA) via complexation. The reversible crosslinkages between catechol and boronate decrease with increasing glucose concentration leading to higher swelling and faster insulin release at hyperglycemia as compared to euglycemia. Such superior glucose‐responsive properties are demonstrated by in vitro analyses and in vivo efficacy studies. The hydrogel polymers also preserve native structure and bioactivity of insulin, attributable to the interaction of hyaluronic acid (HA) with insulin molecules, as revealed by experiments and molecular dynamics simulations. The simplicity in the design and fabrication process, and glucose‐responsiveness in insulin delivery impart the matrix microneedle (mMN) patch great potential for clinical translation.

## Graphical abstract

A self‐crosslinkable and multifunctional polymer‐based matrix microneedle (mMN) patch is developed, which is capable of stabilizing highly concentrated insulin and delivering insulin on demand as demonstrated by in vitro analyses and in vivo efficacy studies. The introduced crosslinking strategy eliminates conventionally used harsh conditions for making crosslinked microneedle (MN) patches and thus enables patch fabrication with encapsulated susceptible protein in one step.

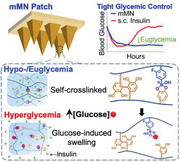

## 1 Introduction

Diabetes is a serious medical condition currently affecting over 463 million people worldwide.^[^ ^1^ ^]^ All people with type 1 diabetes (T1D) and advanced type 2 diabetes require insulin therapy to control their blood glucose levels (BGLs). Improved glycemic control currently relies on hybrid closed‐loop insulin delivery systems^[^ ^2^ ^]^ that combine a continuous glucose monitoring device connected to an insulin pump delivering insulin at high glucose levels (hyperglycemia). However, patients need to wear such devices 24 h a day with tethered pumps and tubing that pose risks, such as skin infections from the cannula, device malfunction, and issues with insulin instability.^[^ ^3^ ^]^ Furthermore, the high cost of the devices and accessories presents a problem of affordability for many patients, while the required patients’ intervention and cumbersome maintenance are often complicated by unpredictable eating and exercise patterns and accompanied by the risk of hypoglycemia.^[^ ^3^, ^4^ ^]^ To overcome these problems, developing glucose‐responsive polymeric systems that regulate glucose levels without a need for patient intervention has gained increasing interest.^[^ ^5^, ^6^, ^7^ ^]^ Among various systems, glucose‐responsive polymeric microneedle (MN) patches have received special attention due to their convenient application, easy removal, and glucose‐responsive release of adequate amounts of insulin at hyperglycemia.^[^ ^8^, ^9^, ^10^, ^11^, ^12^ ^]^ An ideal polymeric MN patch for insulin delivery needs to withhold a high insulin payload, preserve native insulin structure/bioactivity, and rapidly release insulin over extended cycles on demand,^[^ ^13^, ^14^ ^]^ for which hydrogel‐based MN patches show a great potential.^[^ ^15^, ^16^ ^]^ In addition to the tailorable polymer composition, hydrogel‐based MN patches at dry state are able to physically penetrate the stratum corneum, access epidermal interstitial fluid, and allow transdermal delivery of macromolecules.^[^ ^17^ ^]^ However, most of the reported hydrogel‐based MN patches^[^ ^11^, ^15^, ^16^, ^18^ ^]^ require thermal, photo‐ or chemical crosslinking that may cause insulin denaturation.^[^ ^19^ ^]^ Thus, fabrication of insulin‐containing MN patches via protein‐friendly crosslinking methods without using the above‐mentioned crosslinking means needs to be explored.

To date, the majority of glucose‐responsive MN patches employed traditional glucose‐sensing components like glucose oxidase (GOx),^[^ ^11^, ^20^ ^]^ or phenylboronic acid (PBA).^[^ ^21^, ^22^, ^23^ ^]^ However, both systems still face multiple challenges. For example, oxidation of glucose by GOx was found to be very slow in the physiological condition,^[^ ^24^ ^]^ and the values of the most reported boronic acid‐based monomers are mismatched for glucose‐responsive applications.^[^ ^25^ ^]^ Recently, a new charge‐switching mechanism has been utilized to fabricate glucose‐responsive matrix MN patch for insulin delivery with interesting results.^[^ ^16^ ^]^ However, the complex MN fabrication process that involves multiple steps of polymerization and UV crosslinking reaction in the presence of organic solvent and insulin, followed by repeated washing, can be a potential barrier to developing such a device for medical use.^[^ ^26^, ^27^ ^]^ A recently reported dual‐responsive dissolvable MN system incorporated PBA‐containing self‐assembled vesicles that loaded with GOx and insulin to demonstrate rapid insulin release.^[^ ^28^ ^]^ However, lengthy copolymerization processes and additional drug‐loading steps are needed for MN preparation. Therefore, there is still a need for a simpler and more protein‐friendly procedure for MN fabrication to overcome future clinical challenges of MN technology for insulin delivery.

**Figure 1:**
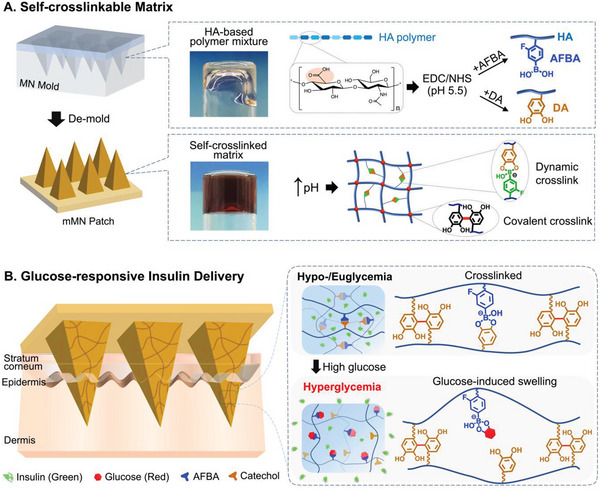
mMN fabrication and its glucose‐responsive insulin delivery. A) Schematic illustration of mMN fabrication and mechanism of self‐crosslinking. The polymer mixture for mMN fabrication is comprised of HA polymer functionalized with AFBA and DA via EDC/NHS coupling reaction. The self‐crosslinked mMN is formed by raising the pH to a slightly alkaline condition. The matrix contains both dynamic and covalent crosslinks within the hydrogel network. Dynamic crosslinking (reversible) is the association between the catechol group of DA and AFBA, whereas covalent crosslinking (irreversible) is attributed to the DA‐DA linkage. B) Mechanism of glucose‐responsive insulin delivery from the mMN patch. Under hypo/eu‐glycemic conditions, the boronate of AFBA forms a complex with catechol, and insulin is physically trapped within the matrix network. In the presence of high glucose, AFBA preferably forms a complex with glucose by dissociating with catechol groups. This uncomplexation breaks the dynamic crosslinked network, which enhances the hydrogel swelling and promotes insulin release.

Herein, we took advantage of mussel‐inspired chemistry and designed a novel self‐crosslinkable polymer‐based matrix MN (mMN) patch capable of encapsulating highly concentrated insulin, with super swelling capability, and rapid delivery of insulin at hyperglycemia. The mMN array is made of a hyaluronic acid (HA) polymeric backbone functionalized with catechol‐containing dopamine (DA) and 4‐amino‐3‐fluorophenylboronic acid (AFBA) that can be quickly crosslinked upon mixing of aqueous solutions of the polymers at slightly alkaline pH.^[^ ^29^, ^30^, ^31^ ^]^ The crosslinking of mMN is formed via i) covalent catechol‐catechol (DA‐DA) linkages^[^ ^32^, ^33^ ^]^ and ii) the dynamic/reversible complexation between catechol and AFBA without the need of an additional crosslinking agent and/or application of heat or UV light (**Figure** **1**A).^[^ ^34^, ^35^ ^]^ The complexation of catechol‐AFBA not only plays a role in the crosslinking process but also provides a glucose‐responsive property to the mMN patch. The latter stems from the glucose level‐dependent complexation between catechol and boronate groups.^[^ ^35^ ^]^ Under physiological and hyperglycemic conditions, AFBA preferably binds to glucose by dissociating with the catechol groups,^[^ ^36^, ^37^ ^]^ leading to the breaking of the secondary crosslinked network, enhancing the swelling of the hydrogel and promoting insulin release (Figure 1B). Conversely, under hypo/normo‐glycemic conditions, AFBA reversibly forms a complex with catechol, reforming its secondary crosslinked network to promote hydrogel densification, thereby retarding insulin release and reducing the risk of hypoglycemia. This novel mMN patch demonstrated sufficient skin penetration, rapid and ultrahigh swelling capability, high insulin loading capacity, and effective hypo/hyper‐glycemia prevention. Owing to its excellent insulin delivery profile at hyperglycemia and capability of preserving the bioactivity of insulin, the proposed MN patch is of great potential for minimally‐invasive administration of insulin, as confirmed via in vivo experiments.

## 2 Results and Discussion

**Figure 2:**
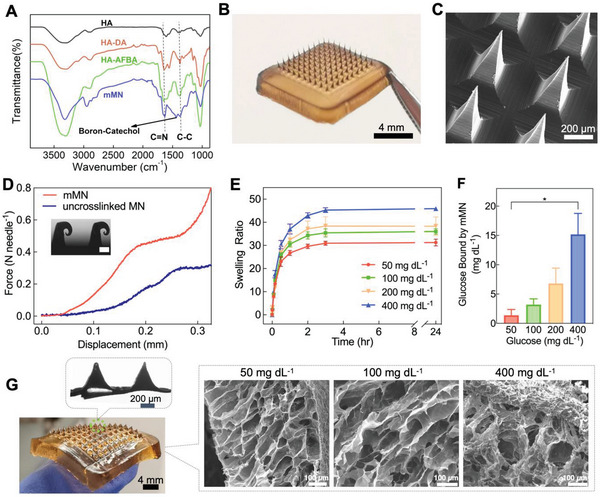
In vitro characterization and glucose‐dependent swelling studies. A) FTIR spectra of HA, HA‐DA, HA‐AFBA, and mMN. B) A photograph of the mMN patch, dimensions of the array: 8 × 8 mm. C) SEM image of the mMN tips. D) Mechanical strength of the mMN patch versus uncrosslinked MN patch; inset: microscopic image of compressed mMN tips (scale bar: 100 µm). E) Swelling ratio of the mMN in pH 7.4 PBS containing various glucose concentrations at 34 °C (*n* = 3). F) Glucose‐binding capability of the glucose‐responsive mMN in PBS at various glucose concentrations (*n* = 3). Statistical significance was calculated using a two‐tailed Student's *t*‐test. ^*^ *p* < 0.01. G) Photograph of the swollen mMN (Top inset: bright‐field micrograph of mMN tips after reaching maximum swelling. Right inset: SEM images of mMN swelled at different glucose concentrations. Data are presented as mean ± standard deviation (SD).

To functionalize the HA polymer, DA and AFBA were conjugated to HA via carbodiimide and *N*‐hydroxysuccinimide (EDC/NHS) coupling reaction to form HA‐DA and HA‐AFBA, respectively (Figure 1A). To confirm the successful conjugation of the functional groups, the modified and purified polymers were characterized using ^1^H‐NMR and UV–vis spectroscopy (Figures S1 and S2, Supporting Information). The degree of conjugation for DA and AFBA was determined to be ≈15.03% and 15.15% respectively. After aqueous solutions of HA‐DA and HA‐AFBA polymers were mixed under slightly alkaline conditions (pH 7.8), the transparent polymer mixture turned into a brown color once a hydrogel was formed. The hydrogel formation is due to the irreversible covalent DA‐DA connection^[^ ^32^, ^38^ ^]^ and reversible complexation between AFBA with catechol groups of DA^[^ ^35^ ^]^ (Figure 1A). Fourier transform infrared (FTIR) spectroscopy was used to confirm the successful crosslinking of the mMN patch (**Figure** **2**A). Compared to HA, HA‐DA, and HA‐AFBA show two peaks at 1591, and 1285 cm^−1^, attributed to C═N stretching vibrations and aromatic C─C stretching vibrations, respectively, indicating that DA and AFBA functional groups were successfully conjugated onto the HA backbone. The IR spectrum of polymers after crosslinking shows a new peak at 1480 cm^−1^ due to the formation of the catechol‐boronate complex.^[^ ^39^ ^]^ Furthermore, a significant reduction in the broadband of hydroxyl groups of catechol at ≈3300–3400 cm^−1^ indicates the formation of covalent DA‐DA linkage.^[^ ^40^ ^]^ The rheology of HA‐DA and HA‐AFBA mixtures at various weight ratios was studied to identify a desired gelation kinetic that is suitable for the patch fabrication while achieving glucose‐responsiveness (Figure S3 and Table S1, Supporting Information). Among the tested ratios, HA‐DA and HA‐AFBA at a 2:1 ratio was selected for fabrication of the mMN patch due to the relatively slower gelation phase, easier transferring of the hydrogel to the mold, and better integrity of formed MNs with sharp tips. As shown in Figure 2B,C, the mMN patch consisted of a 10 × 10 array of microneedles with 800 µm in length and a pyramidal shape. The distinctive brown color of the patch is due to the oxidation of catechol groups that occurs during the covalent DA‐DA crosslinking.^[^ ^38^, ^39^ ^]^ The effect of crosslinking on enhancing mechanical strength of the mMN patch was evaluated by compression stress‐strain testing. The force‐at‐break of the crosslinked mMN patch was over 0.45 N per needle compared with 0.27 N per needle for the uncrosslinked MN patch (Figure 2D), demonstrating that crosslinking provided an adequate mechanical strength for skin penetration in the following animal studies.^[^ ^41^, ^42^ ^]^

The swelling capability of the mMN arrays was examined by immersing the mMN patch in phosphate buffered saline (PBS, pH 7.4) with varying glucose concentrations (50, 100, 200, or 400 mg dL^−1^), representing hypoglycemic, euglycemic, and hyperglycemic conditions. Glucose concentration of 200 mg dL^−1^ was included as a clinically relevant hyperglycemic level for people living with diabetes, whereas 400 mg dL^−1^ is normally observed in T1D rat models. As shown in Figure 2E, the mMN became extremely swollen at hyperglycemia and reached over 45 times of its dry weight after 3 h of immersing in the glucose media at 400 mg dL^−1^ (Figure S4, Supporting Information). It should be noted that the needles of the swellable mMN remained intact at the maximum swelling state (Figure 2G). A similar swelling trend was obtained by observing the changes in the volume of mMN tips after insertion into a glucose‐containing agarose gel (Figures S5 and S6, Supporting Information). Such a high swelling ratio is consistent with previous observations for dynamically associative crosslinked hydrogels.^[^ ^43^ ^]^ The mechanism of glucose‐dependent swelling of the mMN is further supported by a glucose‐binding efficiency study. The amount of glucose bound to mMN through the AFBA groups in hyperglycemic media (400 mg dL^−1^) was 4.8‐fold greater than that in the euglycemic media (100 mg dL^−1^) (Figure 2F). The scanning electron microscopy (SEM) images of the internal microstructures/porosity of the swollen mMN patches have been shown in Figure 2G (inset). As expected, the pore size of the hydrogels increased by increasing the glucose concentration due to the higher swelling capability of the mMN patch at the hyperglycemic condition.

**Figure 3:**
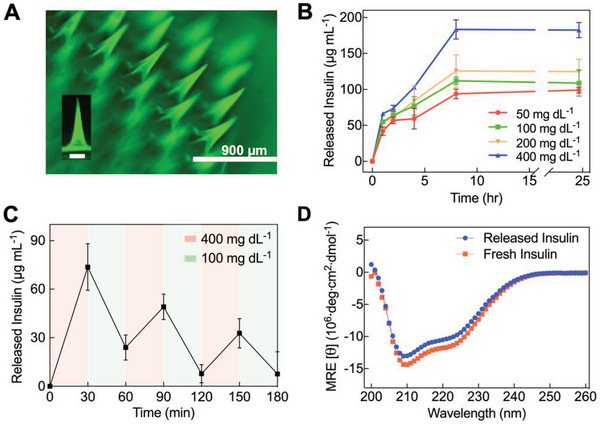
In vitro glucose‐responsive insulin release. A) Fluorescence microscopy image of mMN array loaded with FITC‐Insulin (inset: a magnified image of the microneedle, scale bar: 200 µm). B) In vitro glucose‐responsive release of accumulated insulin from the mMN in PBS with glucose concentration corresponding to hypoglycemia (50 mg dL^−1^), euglycemia (100 mg dL^−1^), clinically relevant hyperglycemia (200 mg dL^−1^), or hyperglycemia often studied in T1D rats/mice (400 mg dL^−1^) (*n* = 3). C) Pulsatile release profile of insulin from mMN patches in alternating glucose media (100 or 400 mg dL^−1^) (*n* = 3). D) Circular dichroism spectra of insulin released from the mMN compared to freshly prepared insulin solution. Data are presented as mean ± SD.

The mMN system showed 100% insulin loading efficiency and a high loading capacity (18.2 wt.%) owing to the direct addition of insulin during mMN formation, providing an advantage for multi‐cycle insulin delivery.^[^ ^44^ ^]^ The even distribution of insulin in the entire mMN tips was evidenced by fluorescence microscopy of the fluorescein isothiocyanate‐labeled insulin (FITC‐insulin) loaded mMN patch (**Figure** **3**A), which renders fast glucose‐responsive insulin release. In vitro insulin release from the mMN patch was examined by immersing the mMN patches in PBS containing various glucose concentrations (50, 100, 200, and 400 mg dL^−1^). As shown in Figure 3B, the rate and extent of insulin release from the mMN at hyperglycemic state (200 and 400 mg dL^−1^) were greater than those at euglycemic (100 mg dL^−1^) and hypoglycemic (50 mg dL^−1^) levels, demonstrating the mMN patch releases insulin in a glucose‐responsive manner. As the glucose concentration increases, more glucose molecules replace catechol groups in the catechol‐AFBA complex, forming more glucose‐AFBA complex and causing more mMN swelling and insulin release. Conversely, when the glucose concentration decreases in the media, the driving force for catechol‐AFBA complexation increases, thereby regenerating the secondary crosslink network while lowering the rate of insulin release in a feedback control loop. As a result, a pulsatile insulin release profile was observed when the patch was immersed alternatively in the euglycemic and hyperglycemic solutions every 30 min for several cycles (Figure 3C). This cyclic high‐low insulin release rate in response to glucose concentrations affords application of the mMN for daily glycemic management. In contrast, non‐glucose‐responsive MN patches (made of HA‐DA without glucose‐responsive unit AFBA) released insulin quickly independent of glucose concentration (Figure S7, Supporting Information). Although an initial burst release of insulin was observed, such an effect would not be significant in vivo as only the tips of the microneedles are inserted in the skin where their swelling would be restricted.

To ensure that the native insulin stability was preserved after the patch fabrication and subsequent release process, the secondary structure of released insulin was determined by circular dichroism (CD) spectropolarimetry. The CD spectra of freshly released insulin from the mMN patch showed two signature bands at 208 and 222 nm, predominantly attributed to the alpha‐helices of insulin (Figure 3D), which were unchanged as compared to fresh insulin.^[^ ^45^ ^]^ The remaining protein content and presence of degradation species of released insulin were analyzed by reversed‐phase high‐performance liquid chromatography (RP‐HPLC). The chromatography peak of released insulin from the patch was similar to the freshly prepared insulin with an elution time of ≈11.3 min, with no detectable degradation peaks (Figure S8, Supporting information). The effect of the polymers on preserving insulin secondary structure was further investigated by MD simulation. Figure S9A,B (Supporting Information) show the percentage of secondary structures versus insulin residue numbers, and Figure S9C,D (Supporting Information) reveal the percentage of secondary structures and residue numbers versus MD simulation time in the absence and presence of the polymer, respectively. The results of MD simulation have good agreement with the experimental results of CD.

**Figure 4:**
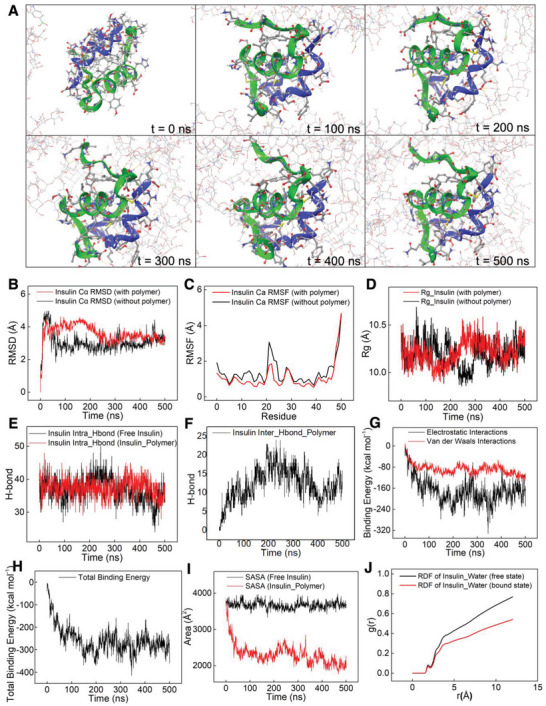
A) Snapshots of the insulin/polymer system at various times, *t*, during the 500 ns MD simulation. Insulin secondary structure is depicted in blue/green ribbons. B–J) Results extracted from MD simulations of insulin in the absence and presence of the polymer. B) Insulin root‐mean‐square deviation. C) Insulin root‐mean‐square fluctuation. D) Insulin radius of gyration in the absence and presence of the polymer. E) Intra‐H‐bonds in insulin in the absence and presence of the polymer. F) Intermolecular H‐bonds between insulin and the polymer. G) Electrostatic and Van der Waals binding energy between insulin and the polymer. H) Total binding energy between insulin and the polymer. I) The solvent accessible surface area of insulin in the absence and presence of the polymer. J) Radial distribution function of water molecules around free insulin and polymer‐bound insulin. The distance r (Å) is the distance between insulin and the first layer of surrounding water molecules.

In addition to the experimental investigations, molecular modeling and computational biology were employed to study the molecular mechanism underlying the stabilization effect of the hydrogel polymer on insulin molecule. The interaction mode between insulin and the functionalized polymers was studied using all‐atom molecular dynamics (MD) simulations for a mixture where the molar ratio of polymer chains to insulin was assigned according to the ratio used experimentally. **Figure** **4**A shows the snapshots of insulin at *t* = 0 (starting point), 100, 200, 300, 400, and 500 ns in the presence of HA‐DA and HA‐AFBA polymers. As shown in Figure 4A, the polymer chains surrounded the insulin molecule and over time, they completely encapsulate the insulin molecule. To understand the effects of the polymer on the structure and stability of the protein, the root mean squared deviation (RMSD) values of insulin in the absence or presence of the polymers were analyzed (Figure 4B). In this study, the RMSD values of the polypeptide alpha carbon (*C*α) atoms were calculated during the simulation with reference to its initial structure. At the beginning of the simulation (*t* = 0 to *t* < 150 ns), the RMSD values of insulin in the presence of polymers were higher than their free state. The higher mobility of the peptide can be attributed to the initial movement of the molecule to interact with the polymer residues. However, at 150 ns, the RMSD decreased, attributable to the stabilized interaction of insulin with the polymer (Figure 4B). The root mean square fluctuation (RMSF) of each residue in the insulin molecule was also monitored to determine the effects of polymer on the dynamic behavior of polypeptide residues (Figure 4C). The computed RMSF values for the Cα atoms in insulin were compared to the B‐factor (thermal factor) of polypeptide atoms extracted from X‐ray crystallography experiments (Figure S9E, Supporting Information).^[^ ^46^ ^]^ A similar trend observed for computed RMSF and B‐factor values validated our MD simulation results. As depicted, all insulin residues are sensitive to the presence of the polymers, as shown by their lower RMSF values in the complex state. This characteristic can be attributed to the favorable interactions between insulin and the functionalized‐HA polymers.^[^ ^47^ ^]^ Figure 4D exhibits typical results for the radius of gyration (*R*_g_) of insulin in the absence and presence of the polymers. It is seen that the protein in the presence of the polymer is nearly unaltered at 300 ns and onward as compared to free insulin, suggesting that the polymer has no remarkable effect on the *R*_g_ of insulin.

Previous studies have demonstrated that hydrogen bonds (H‐bonds) have a pivotal role in the complex formation between polymer and protein molecules.^[^ ^48^ ^]^ Thus, the intra‐H‐bonds in insulin molecule and inter‐H‐bonds between insulin and the polymers were studied. The average number of intra‐H‐bonds in both free and bound insulin remained unchanged at an average value of 38 (Figure 4E), while the average inter‐H‐bonds between insulin and the polymer during the simulation were found to be 12.3 (Figure 4F). This hydrogen bonding pattern may be explained as that the protein is stabilized by HA‐based polymers that shield the protein. The donor and acceptor atoms involved in the intermolecular H‐bonding are summarized in Table S2 (Supporting Information). Residues Ile2 (chain A), Cys7 (chain B), Thr8 (chain A), Ser12 (chain A), Glu13 (chain B), Leu17 (chain B), Tyr19 (chain A), Asn21 (chain A), Pro28 (chain B), Lys29 (chain B), and Ala30 (chain B) from insulin participate in H‐bonding with the functionalized‐HA polymers. It has been found that Asn21, as a conserved residue, plays a key role in the deamination of the protein in an acidic medium,^[^ ^47^ ^]^ and in maintaining the specific spatial configuration of insulin required for its bioactivities.^[^ ^49^ ^]^ Because Asn21 residue is located at the end of the chain, its H‐bonding with the polymer may stabilize insulin structure and maintain insulin bioactivity. On the other hand, H‐bonding of backbone residues Leu17 (chain B) and Ile2 (chain A) with the polymer could block insulin fibrillation, because the point mutation of Leu17 (chain B) could delay the lag phase of insulin fibrillation,^[^ ^50^ ^]^ and Ile2 (chain A), a hydrophobic residue, acts as a nucleation‐prone residue in insulin fibrillation.^[^ ^50^ ^]^

In addition to hydrogen bonding, two major types of free energy of binding (electrostatic interaction and van der Waals) and total binding energy between insulin and the polymer were also computed. As shown in Figure 4G, electrostatic interaction between insulin and the polymer is more significant than van der Waals’ interactions. The average total binding energy of insulin with the polymers is −258.2 kcal mol^−1^ (Figure 4H). Figure 4I shows that the average solvent accessible surface area (SASA) of insulin is reduced from 3674.15 Å^2^ for free insulin to 2319.33 Å^2^ for insulin with the polymers, which can be ascribed to the “shielding effect” of the HA‐based polymers. As discussed above and reported previously,^[^ ^51^, ^52^ ^]^ the high hydrophilic nature of the polymer could provide a stabilization effect, and the shielding effect via encapsulation by ionically charged HA could prevent insulin aggregation. The result suggests that the functionalized‐HA polymers can possibly prevent insulin fibrillation and proteolysis.

In addition to protein fibrillation, random interactions with water molecules also cause insulin deactivation.^[^ ^53^ ^]^ Our simulation results showed that the density of radial distribution function (RDF) of water molecules around the insulin was reduced in the presence of the polymers as compared to free insulin (Figure 4J). In other words, in the presence of the polymers, more water molecules are excluded from the surface of the insulin, which can decrease the possibility of random interaction of insulin with water molecules. However, the height of the first peak of RDF at the distance smaller than 3 Å related to the first layer of bound water molecules shows the same value for free insulin and polymer‐insulin (Figure 4J).

**Figure 5:**
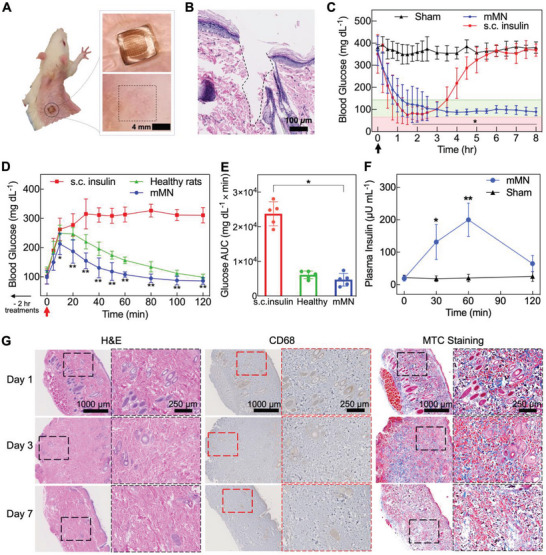
In vivo evaluation of mMN patch in an STZ‐induced type 1 diabetic rat model. A) Photograph of a rat dorsum skin transcutaneous treated with the mMN patch (right and top left) and 12 h‐post mMN application (bottom left). B) H&E‐stained tissue section of the rat skin penetrated by mMN, scale bar = 100 µm. C) In vivo insulin efficacy of mMN patches versus sham patches (no insulin‐loaded mMN patch) and subcutaneous (s.c.) insulin injection (0.5 U kg^−1^) (*n* = 5). Rats were fasted for 5 h prior to experiments and treatments occurred at *t* = 0 min. The green region indicates normoglycemia, and the red region indicates hypoglycemia. The black arrow indicates the time of microneedle patch administration. The mMN and sham were compared for statistical significance and determined by a two‐way repeated measures ANOVA and Bonferroni multiple comparisons test. ^*^ *p*‐value < 0.001. D) intraperitoneal (i.p.) glucose tolerance test (IPGTT) in diabetic rats 2 h post‐administration of mMN patch compared with healthy rats and 2 h post s.c. insulin injection as control (0.5 U kg^−1^) (*n* = 5). The red arrow indicates the time of i.p. injection of glucose (2 g kg^−1^), which was 2 h after the treatments. Statistical significance between groups treated with mMN and s.c. insulin was determined by a two‐way repeated measures ANOVA with Bonferroni multiple comparisons test. ^*^ *p*‐value < 0.05, ^**^ *p*‐value < 0.001. E) The area under the curve (AUC) of blood glucose concentration profiles during IPGTT with the baseline set to blood glucose level at 0 min. Statistical significance was calculated using a two‐tailed Student's *t*‐test. ^*^ *p*‐value < 0.001. F) Plasma insulin levels at *t* = 0 min (before glucose challenge), *t* = 30, 60, and 120 min (after glucose challenge) as determined by insulin ELISA (*n* = 5). Statistical significance between groups was determined using a two‐tailed Student's *t*‐test. ^*^ *p*‐value < 0.01, ^**^ *p*‐value < 0.001. G) H&E‐stained (left) and CD68‐stained (middle) and Masson's trichrome (MTC, right) staining results. The treated rat skin tissue was harvested on days 1, 3, and 7 post‐mMN patch removals. Inset images with black/red box sections are enlarged views of the box sections on the left. Data are presented as mean ± SD.

For the in vivo study, the successful skin penetration capability of the mMN patch was first confirmed by the delivery of trypan blue dye into the rat skin (Figure S10, Supporting Information) followed by hematoxylin & eosin (H&E) staining histology study (**Figure** **5**A,B). Due to its minimal invasiveness and high biocompatibility, the mMN traces gradually disappeared post‐patch application (Figures S10B and S11, Supporting information). To investigate the in vivo efficacy of the mMN patches against hyperglycemia, streptozotocin (STZ)‐induced T1D male Sprague–Dawley rats were randomly grouped and treated with either insulin‐loaded mMN patch (58 U), sham patch (as a control), or subcutaneous insulin injection (0.5 U kg^−1^). All T1D diabetic rats were fasted for 5 h before the study began. The mMN patches, sham patches, or insulin injections were administered to the rats at *t* = 0, and their BGLs were monitored for 8 h. As shown in Figure 5C, the BGLs in the control groups treated with sham patches remained at hyperglycemia throughout the study. However, the BGLs of the rats treated with the mMN patch and insulin injections rapidly decreased and fell below 165 mg dL^−1^ within 1 h. The rats treated with insulin injection did not remain in the normoglycemic state and their BGLs gradually increased to hyperglycemia after 3.5 h. Conversely, the mMN patch successfully regulated BGLs of the rats within a tight glycemic target range (<190 mg dL^−1^ and >70 mg dL^−1^) for >7 h, which is longer than matrix MN patches reported by other research groups.^[^ ^16^, ^54^, ^55^, ^56^, ^57^ ^]^ Unlike the glucose‐responsive mMN patch, non‐glucose‐responsive MN patches loaded with the same amount of insulin (58 U) but with no AFBA units released insulin rapidly and indifferently of glucose concentration (Figure S7, Supporting Information). Therefore, they could not maintain normoglycemia for an extended period and the blood glucose in the treated rats quickly increased after 3 h (Figure S12, Supporting Information).

To evaluate the in vivo glucose control capability and risk of hypoglycemia of the “smart” mMN patch, healthy rats were treated with the mMN patch compared to the non‐glucose‐responsive MN patch. The results showed that the rats treated with non‐glucose‐responsive MN experienced a significant decrease in glucose levels (Figure S13A, Supporting Information). Conversely, the mMN treatment group did not experience a significant reduction in glucose. The corresponding hypoglycemia index,^[^ ^11^ ^]^ quantified by the difference between the initial glucose levels to the nadir in each experiment divided by the time the nadir occurred in that experiment, suggested that the mMN patch had a significantly lower hypoglycemia index compared to the non‐glucose‐responsive patch (Figure S13B, Supporting Information).

To study the glucose regulation capability of the mMN patch, intraperitoneal glucose tolerance tests (IPGTT) were conducted by injection of 2 g kg^−1^ of glucose 2 h‐post mMN application or insulin injection to lower the initial plasma glucose level to a similar normoglycemic level. As illustrated in Figure 5D, the BGLs in the T1D rats treated with mMN returned to normoglycemic state after the glucose levels peak similar to the healthy rats. In contrast, the T1D rats 2 h after subcutaneous (s.c.) insulin injection experienced sustained hyperglycemia after the glucose challenge. The glucose area under the curve (AUC), a measure of sustained glucose exposure for 120 min, in the s.c. insulin‐treated rats were fivefold that in the mMN patch‐treated rats (Figure 5E). The glucose regulatory effect of the mMN patch can be ascribed to its glucose‐dependent insulin release. As shown in Figure 5F, at *t* = 0 (i.e., 2 h‐post mMN application) when the BGL returned to euglycemia, the plasma insulin in the mMN patch‐treated rats was at a low level like that in the sham patch‐treated group, indicating undetectable insulin was released from the insulin mMN patch at this point. After the glucose challenge at *t* = 0, the plasma insulin level increased dramatically by up to 9.8‐fold at 30 and 60 min and then dropped to the basal level again at 120 min, while no change was found in the sham patch‐treated rats (Figure 5F). These results indicate that the mMN patch is able to adjust the insulin release rate according to blood glucose levels, thus maintaining euglycemia for an extended time without causing hypoglycemia and rapidly suppressing hyperglycemia. Owing to the limitation of the duration of fasting the T1D rats and the number of blood samples that can be taken from each rat in a continued study, the longer‐term efficacy of the insulin mMN patch will be evaluated using a large animal model, which is being pursued by our group.

To examine the biocompatibility, the in vitro cytotoxicity of the patch materials was evaluated on mouse fibroblast cells (NIH‐3T3) and human keratinocyte cells (HaCaT) using MTT assay. As shown in Figure S14 (Supporting Information), due to the high biocompatibility of the HA‐based polymers, no significant toxicity was observed. To further ensure the safety of our patch, in vivo biocompatibility studies were also examined after the mMN patch application on rat skin for 1, 3, and 7 days by analyzing hematoxylin & eosin (H&E), CD68, and Masson's Trichrome (MTC) staining (Figure 5G). From the H&E staining results, slight inflammatory neutrophil infiltration was observed 12 h after mMN application on day 1, which was recovered on day 7. CD68 staining results showed the presence of macrophages at the site of perforation of rat skin treated with mMN patch were minimal by day 7, which was comparable with healthy rats without treatment (Figure S15, Supporting Information). Lastly, MTC staining results appeared similar between the treatment and control groups, demonstrating no significant changes to the collagen and extracellular matrix structure of the perforated rat skin across 7 days after patch application. Overall, histological analysis across all types of staining indicated minimal evidence of cell inflammation, signs of tissue damage, and skin structural deformation post‐patch applications.

## 3 Conclusion

In summary, we have fabricated a self‐crosslinkable polymeric mMN patch capable of encapsulating and delivering insulin at hyperglycemia, as well as preserving the native structure/bioactivity of the peptide via the use of biocompatible HA and protein‐friendly crosslinking conditions. The introduced crosslinking mechanism for MN fabrication eliminates the need for conventional harsh crosslinking methods like heating or irradiation. The simple pH adjustment of the polymer solution for making crosslinked mMN eliminates multistep processes of fabrication and brings opportunities for scale‐up of MN patch manufacturing. The MD simulation results suggest that the native insulin structure can be preserved via the interactions among insulin, functionalized‐HA polymers, and water molecules. Glucose‐responsive properties of the mMN were demonstrated in vitro and further verified on a T1D rat model with the glycemic target maintained for at least 8 h.

Although continuous subcutaneous insulin infusion provides vastly superior glycemic control than insulin injections, the cost and inconvenience of the system prevent its widespread use. In contrast, a delivery strategy using a “smart” mMN patch could potentially improve the quality of lives of people with diabetes by offering them convenient, painless, and affordable glycemic control. The simplicity in the design and fabrication process offers the mMN system promising potential for scale‐up and clinical translation. The mMN patch developed in this study has successfully demonstrated its application as a glucose‐responsive, disposable transdermal delivery platform that could be optimized for daily use. The described system can also be implemented for the delivery of other therapeutic hormones, such as insulin analogs^[^ ^12^ ^]^ and glucagon,^[^ ^23^ ^]^ for achieving long‐term glycemic management. To fully realize the potential of the mMN patch for translation, optimization of the design of the patch and manufacturing process is needed. Lastly, findings from the computational modeling of the binding between insulin and polymers demonstrate the utility of MD simulations in elucidating the underlying mechanisms of protein stabilization by selected polymeric materials.

## 4 Experimental Section

Details of the materials and experimental methods are available in the Supporting Information.

### Statistical Analysis

All results are presented as mean ± standard deviation. Statistical analysis was performed using a two‐tailed Student's *t*‐test to analyze the difference between the two groups. Differences between experimental groups and control groups were considered statistically significant with a *p*‐value < 0.05. Statistical significance with multiple comparisons in the in vivo insulin efficacy and in vivo IPGTT was determined using a two‐way repeated measures analysis of variance (ANOVA) with a Bonferroni multiple comparisons post‐hoc test.

## Conflict of Interest

The authors declare no conflict of interest.

## Supporting information

Supporting Information

## Data Availability

The data that support the findings of this study are available from the corresponding author upon reasonable request.
