# Supplementary material for: “Smart” Matrix Microneedle Patch Made of Self‐Crosslinkable and Multifunctional Polymers for Delivering Insulin On‐Demand
Source: Adv Sci (Weinh). 2023 Sep 18;10(30):2303665. doi: 10.1002/advs.202303665 (PMC10602565; doi:10.1002/advs.202303665)
Supplement: Supplementary file 1 — Supporting Information [file ADVS-10-2303665-s001.pdf]

ADVANCED SCIENCE
Open Access

Supporting Information

for *Adv. Sci.*, DOI 10.1002/advs.202303665

"Smart" Matrix Microneedle Patch Made of Self-Crosslinkable and Multifunctional Polymers for Delivering Insulin On-Demand

*Jackie Fule Liu, Amin GhavamiNejad, Brian Lu, Sako Mirzaie, Melisa Samarikhalaj, Adria Giacca and Xiao Yu Wu**

Supporting Information

"Smart" Matrix Microneedle Patch Made of Self-crosslinkable and Multifunctional Polymers for Delivering Insulin On-demand

Jackie Fule Liu[1,a], *Amin GhavamiNejad*[1,a], *Brian Lu*[1], *Sako Mirzaie*[1], *Melisa Samarikhalaj*[2], *Adria Giacca*[2], *Xiao Yu Wu*[1,*]

[1] Jackie Fule Liu, Dr. Amin GhavamiNejad, Brian Lu, Dr. Sako Mirzaie, Prof. Xiao Yu Wu
Advanced Pharmaceutics and Drug Delivery Laboratory
Leslie L. Dan Faculty of Pharmacy
University of Toronto
Toronto, M5S 3M2, Canada

[2] Melisa Samarikhalaj, Prof. Adria Giacca
Department of Physiology, Faculty of Medicine
University of Toronto
Toronto, M5S 1A8, Canada

Correspondence to: sxy.wu@utoronto.ca

1. Experimental Details

Materials

All of the chemicals were purchased from Sigma-Aldrich (USA) unless specified. Hyaluronic acid (HA, MW 300 kDa) was a gift from Bloomage Biotechnology Corporation Limited (China). USP-grade human recombinant insulin was purchased from Wisent (Quebec, Canada). HPLC-grade acetonitrile and water were purchased from Caledon Labs (Ontario, Canada). The distilled deionized (DDI) water was prepared from a Millipore Purification system. All reagents were of analytical grade or higher and used without further purification.

Synthesis of HA-DA and HA-AFBA

HA conjugated with dopamine (HA-DA) was synthesized using N-(3-dimethylaminopropyl)-N'-ethylcarbodiimide hydrochloride and N-hydroxysuccinimide (EDC/NHS) coupling method.[1] In brief, HA (0.5 g) was completely dissolved in DDI water (50 mL), and the pH was adjusted to 5.5 with 1 M HCl. EDC (1.5 mmol) and NHS (1.5 mmol) were added to the HA mixture separately with a 15 min interval while stirring. After 30 min, dopamine hydrochloride (1.6 mmol) was added to the solution. The pH of the solution was maintained at 5.5. After 12 hr of reaction, the product was purified by dialysis (MWCO 12-14 kD, Spectrum™ Spectra/Por™) at room temperature in DDI water (pH 6) for 3 days with DDI water changed daily. The complete removal of free dopamine was confirmed by UV-vis spectroscopy. The purified product was lyophilized and stored at 4 °C (yield: 96%). Synthesis of HA-AFBA (4-amino-3-fluorophenylboronic acid) was similar to HA-DA. Instead of dissolving HA in DDI water, AFBA (0.6 mmol) was added to the HA mixture (50 mL) containing water/ethanol (70/30 w/w). The HA-AFBA product was purified in DDI water and lyophilized for later use (yield: 80%). The chemical structure of HA-DA and HA-AFBA were

characterized using ^{1}H-NMR in D_2O (400 MHz, MercuryPlus, Varian) and UV-vis spectroscopy.

Fabrication of the Matrix MN Patch

The matrix microneedle patch (mMN) patch was fabricated based on the catechol oxidation method and performed at room temperature. In brief, polymer mixture (50 mg mL^{-1}) was prepared by dissolving a ratio (w/w) of HA-DA and HA-AFBA in DDI water. Lyophilized insulin was solubilized in 0.1 M NaOH solution and thoroughly mixed into the polymer mixture. The amount of solvent used in insulin solution was factored into the polymer and solvent ratio. The pH of the polymer mixture was raised from 6.5 to 7.8. Upon drastic color change, the mixed gel was cast into a polydimethylsiloxane (PDMS) microneedle (MN) mold. Afterward, the mold was placed in a vacuum under 25 mmHg for 5 min to remove trapped air. The crosslinked gel was left air dry at room temperature, and the dried mMN patch was separated from the mold and stored in a desiccator until use. The crosslinked non-glucose responsive MN as a control was prepared the same way as mMN using HA-DA in the absence of HA-AFBA. The uncrosslinked MN patch was prepared similarly but without the pH adjustment.

Characterization of HA-based Polymers and mMN Patch

The gelation kinetics of the polymers for mMN patch fabrication were measured by a hybrid rheometer (Discovery HR3, TA instruments) in a time sweep mode at 1 Hz. In this rheometric analysis, polymer mixtures prepared at various ratios (w/w) of HA-DA and HA-AFBA were adjusted to pH 7.8 for gelation. After complete mixing of the samples, storage modulus (G') and loss modulus (G'') with increasing time were recorded using a 40 mm parallel plate at 25°C. In addition, the bonding configuration of the polymer samples and mMN were identified by attenuated total reflectance (ATR) - Fourier transform infrared (FTIR) spectroscopy. The IR spectra were recorded at room temperature using a Paragon 1000

spectrometer (Perkin Elmer) equipped with VeeMax II variable angle ATR accessory. The dimension and surface morphology of mMN patch was determined by an inverted fluorescence microscope (IX 71, Olympus) and scanning electron microscopy (SEM, Prisma™ E) at an acceleration voltage of 20 kV. For the glucose binding study, mMN patches were incubated in release media at various glucose concentrations (50, 100, 200 or 400 mg dL^{-1}) at 34 °C (skin temperature) for 4 hr. After removing samples from the media, the glucose concentrations were measured using a glucometer (Contour® NEXT ONE, Bayer). The glucose concentrations were calculated based on a glucose standard curve.

Mechanical Strength

The mechanical strength of mMN patches was determined by compression test using Instron 3366 universal testing machine with a compression load cell. The MN array (tips facing upwards) was placed flat on a compression plate. A vertical force was applied perpendicularly at a constant speed of 0.5 mm min^{-1} to the MN patch using a flat-head stainless steel probe. The displacement was measured until the MN tips began to buckle. The initial distance between the base of MN patch and the flat head of the probe was set at 2 mm, with a cell loading capacity set at 10 N. Instantaneous load (force; N) and displacement (distance; mm) were recorded by the testing machine every 0.05 s to generate the load-displacement curve. The force-at-break was recorded as the needle began to buckle.

Characterization of the Swelling Kinetics of mMN Patch

The swelling capability of mMN patch was determined by immersing mMN in pH 7.4 PBS containing varying glucose concentrations (50, 100, 200 or 400 mg dL^{-1}) at 34 ˚C. The net weight of the swelled mMN patch was carefully measured at predetermined time points. The swelling ratio was calculated based on a formula of the weight of the mMN patches at various time (W_t) to its initial net weight (W_0) as shown below.

$$Swelling\ Ratio = \frac{W_t - W_0}{W_0}$$

The swelling kinetics of mMN tips were evaluated by inserting the MNs into a translucent agarose gel (1.4 wt%). The subsequent volume change of the mMN tips with respect to the time of exposure to agarose gel was recorded by a microscope equipped with a CCD camera. The volume change of mMN tips was determined using ImageJ software. The swelling ratio was calculated based on a formula of the volume of the mMN tips at a different time (V_t) to its initial volume (V_0) as shown below. The internal structure of swollen hydrogels was cryo-dried and lyophilized before being examined by SEM.

$$Swelling\ Ratio = \frac{V_t - V_0}{V_0}$$

***In vitro* Release Study**

To verify an even distribution of loaded insulin in the mMN, fluorescein isothiocyanate-labeled insulin (FITC-insulin, 0.1 mg) was loaded into the patch. Glucose-responsive insulin release from mMN patches was determined by incubating the mMN patch in release media at 34 °C with shaking. The release media contains PBS buffer (pH 7.4) and varying glucose concentrations (50, 100, 200 or 400 mg dL^{-1}). Media containing released insulin was quantified by a Pierce™ Coomassie plus protein assay at 595 nm using a BioRad UV-vis plate reader. The amount of released insulin was calibrated with an insulin standard curve. The pulsatile release profile of the mMN patches was analyzed by incubating mMN patches in release media containing 400 mg dL^{-1} glucose for 30 min. The media was then removed and replaced with fresh media containing 100 mg dL^{-1} glucose for another 30 min. This cycle was repeated for 3 hr, and the released insulin was measured using the same protein assay described earlier. Similarly, *in vitro* insulin release from non-glucose responsive MN patches was investigated under the same condition as described previously.

Reversed-phase high performance liquid chromatography (RP-HPLC)

RP-HPLC was used to evaluate the remaining protein contents of released insulin from mMN compared with insulin solution. The insulin solution was prepared freshly and assayed together with the samples. Insulin and any potential degradation products were separated with a C-18 column (Gemini NX C18 - 3 μm 110Å, 2.0 mm × 50 mm, Phenomenex, CA, USA). For all separations, a diluted sample (40 μL) was injected into the column with a flow rate of 0.5 mL min^{-1}. Mobile phase consists of solvent A (0.1% v/v TFA in water) and solvent B (0.1% v/v TFA in acetonitrile). A linear gradient from 15% B to 65% B over 10 min, subsequently with a linear gradient from 65% B to 15% B over 10 min, was used for all separation. The column was washed using 85% A and 15% B for 10 min between samples. Elution times of insulin and any potential degradations were monitored by a UV detector at 215 nm wavelength. The column was maintained at 34 °C throughout the experiment.

Circular Dichroism (CD) Spectropolarimetry

CD spectropolarimetry in the far-UV wavelength region was used to characterize any protein secondary structure deviations of insulin. Insulin released from mMN was collected and measured using a spectropolarimeter (Jasco J-810, MD, USA) equipped with a Peltier temperature controller set to 25 ˚C. Samples were filtered, diluted, and transferred to a 1 cm path length quartz cuvette for far-UV measurements. Samples were scanned at 1 nm intervals between 200 and 260 nm using an 8 sec response time. The measurement was repeated three times and averaged. Sample spectra were compared with the spectrum of standard fresh insulin solution.

Molecular Dynamics (MD) Simulations

For simulating the effect of HA-functionalized polymer on the stability of insulin, a hormone molecule with the PDB ID of 4INS was downloaded from the protein data bank.[2] Overall,

two independent systems (in the absence or the presence of the polymer) were introduced to the MD studies by Desmond package from Schrödinger Inc.[3] For simulating a 200-disaccharides polymer, 10 chains of HA, each containing 20 disaccharide units were constructed. The disaccharide units were HA (76%), HA-DA (16%), and HA-AFBA (8%), which were randomly integrated into each chain. Both systems, free insulin or in complex with the polymer, were solvated in explicit TIP3P (Three-site transferrable Intermolecular Potential) water model and the OPLS3 (Optimized Potentials for Liquid Simulations version 3) force field parameters.[4] A temperature of 310 K, pH of 7.4, and a pressure of 1 bar with the simulation length of 500 ns were assigned for each MD run. The particle-mesh Ewald method was employed to calculate the long-range electrostatic interactions.[5] The cut-off radius for computing the Coulomb interactions was 9.0 Å[6] and a cubic periodic box with periodic boundary conditions was defined for both systems in the solvation step.[7] For neutralizing each system, Na^+ and Cl^- counter ions were added. The distance of 10.0 Å was assigned between the periodic boundary conditions and the closest free insulin or insulin/polymer atom.[6] For each system, the Martyna-Tuckerman-Klein chain coupling scheme.[8] and Nosé–Hoover chain coupling scheme[9] were engaged for the pressure and temperature control during MD simulation, respectively. A total of 1000 frames per MD run were allotted, and the trajectories were saved at 9.6 psec intervals for further analysis. The effect of the polymers on maintaining insulin secondary structure was further investigated by MD simulation.

Animal Experiment

All conducted animal experiments strictly adhered to the ethical and legal requirements of the Ontario Animals for Research Act and the Federal Canadian Council on Animal Care guidelines and were approved by the University Animal Care Committee of the University of Toronto. The *in vivo* evaluations of mMN patches were studied in rats. Male Sprague-Dawley rats (Charles River) were treated with streptozotocin (STZ, 65 mg kg^{-1}) to obtain a

type 1 diabetes (T1D) model. The rats receiving STZ were carefully monitored for 1 week during which time the blood glucose was measured in 2-day intervals using a glucose meter (OneTouch® Ultra®, LifeScan, Inc., USA). Diabetic rats with blood sugar stabilized above 17 mmol L^{-1} were selected for the study. Before the experiment, the rats were fasted for 5 hr and were grouped randomly.

Skin Penetration and Histology

A trypan blue-loaded mMN was applied onto the shaven back of the T1D rats for 15 min. Afterward, the patch was removed, and the treated skin was photographed. For histological analysis, the penetrated skin samples were fixed in 10 % buffered formalin for 24 hr and embedded in paraffin. Samples were then sectioned into 5 mm thick slices and stained with hematoxylin & eosin (H&E).

***In vivo* Insulin Efficacy Study**

Fasted diabetic rats were treated with either insulin-loaded "smart" mMN (58 U), sham patch (as a control), s.c. insulin injection (0.5 U kg^{-1}), or insulin-loaded non-glucose responsive MN (58 U). Rat dorsal skin was shaved, treated with hair removal cream, and dried prior to patch applications. Blood glucose was monitored using the tail-pricking method every 5-15 min. The insulin-loaded non-glucose responsive MN on fasted diabetic rats was used as a control. The 5 hr fasted healthy rats were used to study the risk of hypoglycemia by treating them with insulin-loaded "smart" mMN and insulin-loaded non-glucose responsive MN.

***In vivo* Glucose Tolerance Test**

Intraperitoneal glucose tolerance test (IPGTT) was performed to evaluate the glucose responsiveness of the mMN patch. Briefly, fasted diabetic rats were treated with either s.c. insulin injection (0.5 U kg^{-1}) or insulin-loaded mMN patch. After 2 hr of treatments at t = 0, 50 % dextrose solution (2 g kg^{-1}) was injected intraperitoneally into all rats. The glucose level

was monitored every 5-20 min with a glucometer. To determine plasma insulin levels in diabetic rats, 200 μL of blood samples were drawn from the tail vein of rats at predetermined intervals. The serum was isolated and stored at -20 °C until assay. The plasma insulin concentration was measured using a Mercodia human insulin ELISA kit.

Evaluation of Biocompatibility

The *in vitro* cytotoxicity study of the mMN patch was measured by conducting 3-(4,5)-dimethylthiahiazo(-z-y1)- 3,5-di phenytetrazoliumromide (MTT) assay on both NIH/3T3 fibroblast cells and HaCaT Human keratinocyte cells to mimic skin and tissue. Briefly, both cell types were seeded on tissue-treated 96-well plates at 10,000 cells per well. After 24 hr incubation in Dulbecco's Modified Eagle Medium (200 μL) with 10 % fetal bovine growth serum, cells were treated with samples and incubated for 24 hr at 37°C. After that, MTT reagent (100 μL) was added to each treated well and incubated for 4 hr, followed by adding 10% SDS in 0.01 M HCl (100 μL) to each well. After incubating for another 4 hr, the absorbance of the plate was read at 570 nm using a BioRad UV-vis plate reader. The percent survival was determined using the following equation and plotted on a semi-log scale.

$$\%\, survival = \frac{sample\ sigmal - background\ signal}{control\ sigmal - background\ signal} \times 100$$

Histology Study

The mMN patch was applied to shaved T1D rat skin for 12 hr. After day 1, day 3, or day 7 post-patch removals, the treated skin tissue and healthy skin without patch treatment were harvested and fixed in 10% buffered formalin for 24 hours, embedded in paraffin and sectioned into 5 mm thick slices. Then, the samples were stained with H&E, CD 68, and Masson's Trichrome (MTC).

2. Supplementary Figures

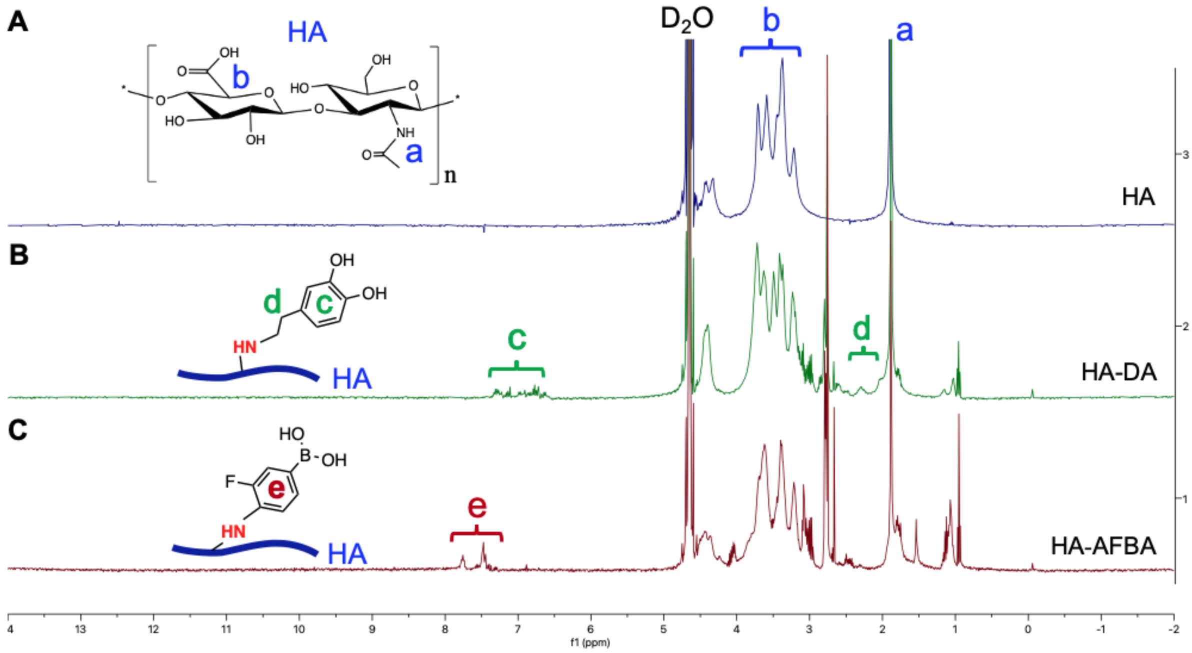

Figure S1. ^{1}H-NMR spectra of HA, HA-DA, HA-AFBA, and their corresponding chemical structure. ^{1}H-NMR (D_2O, 400 MHz, δ ppm). (A) The N-acetyl peak of HA appeared at 1.9 ppm, and multiplets from 3 to 3.8 ppm were associated with disaccharide units in the HA backbone.[10] (B) The chemical shift at δ = 6.7 to 7.32 ppm corresponds to the catechol aromatic ring of dopamine.[11] (C) The multiplets from δ = 7.29 to 7.8 ppm corresponded to the benzene ring of AFBA.[12]

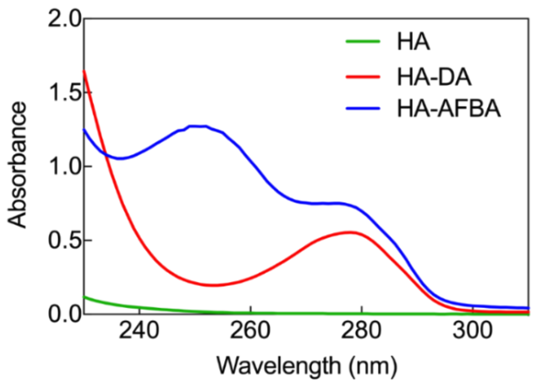

Figure S2. UV absorbance of HA-DA and HA-AFBA conjugates versus HA.

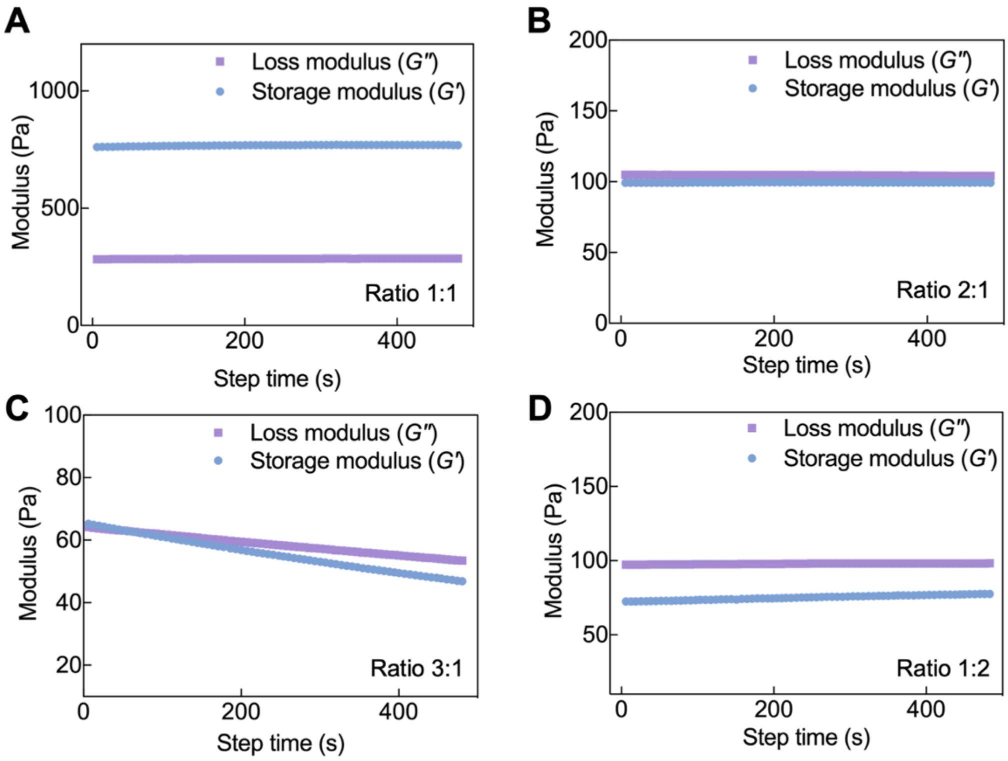

Figure S3. Rheometric analysis of matrix hydrogel formed with various weight ratios of HA-DA and HA-AFBA, respectively, in a time sweep mode after gelation induction.

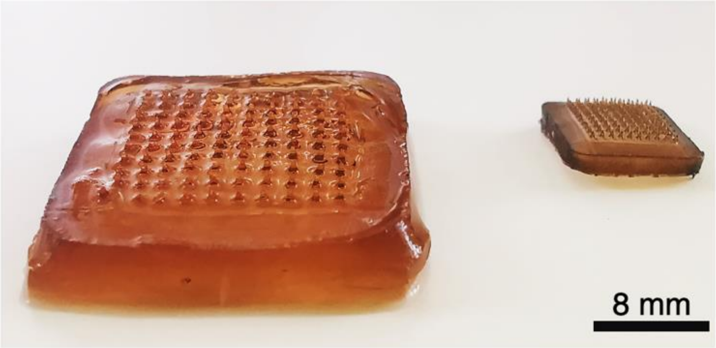

Figure S4. Photographs of a fully swollen mMN patch after 24 hr of media incubation (left) and a dry mMN patch (right).

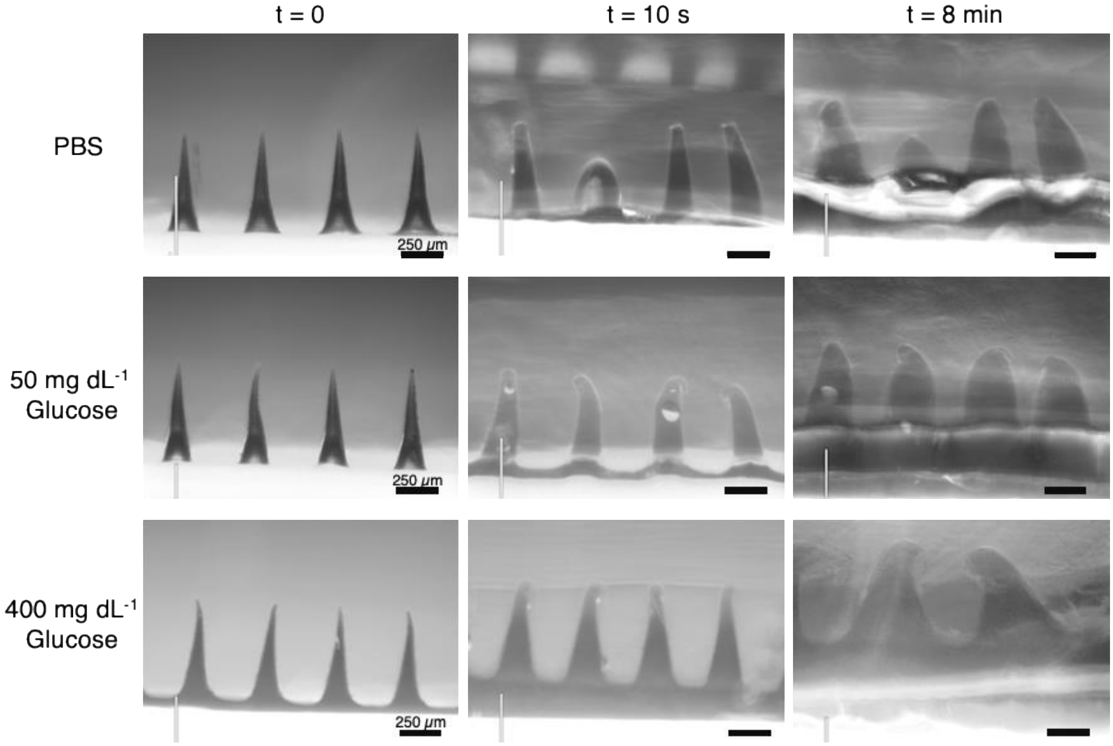

Figure S5. Images of the microneedles swelling profile for Figure S5. The microneedles were inserted into a 1.4% wt agarose hydrogel containing PBS with either 0, 50 or 400 mg dL^{-1} glucose. The swelling volume kinetics were accessed by measuring the micrographs captured at predetermined time points via ImageJ. The images of swelling kinetics at t = 0, t = 10 s, and t = 8 min (final swelling) are illustrated in this figure.

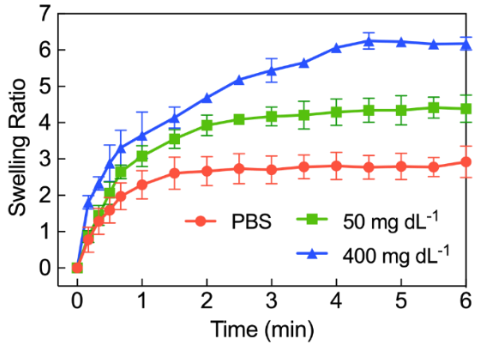

Figure S6. Glucose-dependent swelling profile of the mMN tips determined by the volume change of MNs prior to and at the predetermined time points after inserting the needles into a 1.4 wt% agarose hydrogel. The data presented as mean ± standard deviation (n = 3 and n = 2 for data point of 400 mg dL^{-1} after 2 min due to the microneedles swelled beyond the microscope lens).

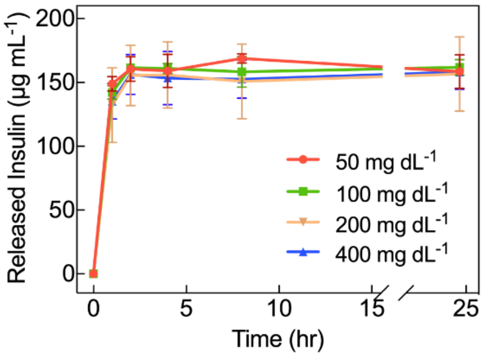

Figure S7. *In vitro* insulin release from non-glucose responsive MN patches in PBS with various glucose concentrations (50, 100, 200, and 400 mg dL^{-1}) (n=3). The MN patch released insulin indifferently at various glucose levels due to the absence of the glucose-responsive monomer unit of AFBA.

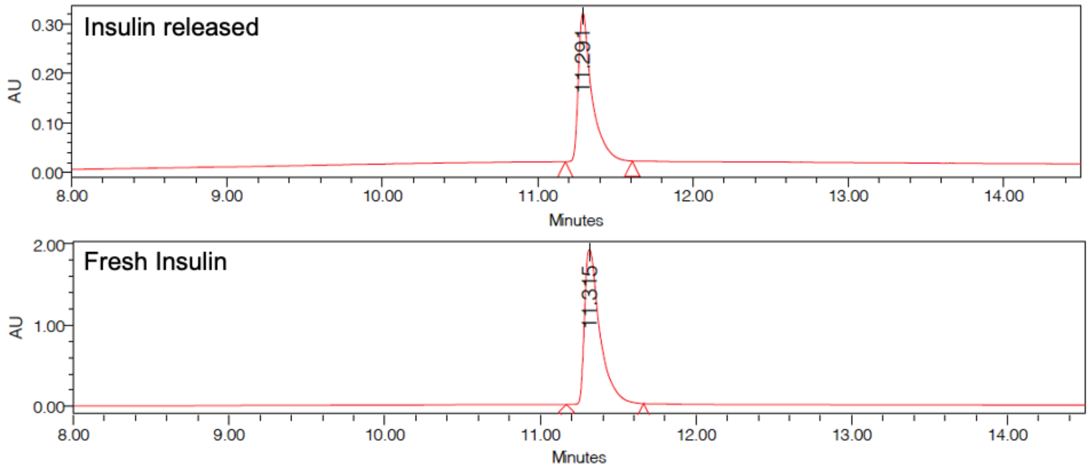

Figure S8. Representative HPLC results of released insulin from mMN and freshly prepared insulin. The time of insulin elution was ~11.3 minutes using a gradient method. No degradation peaks were observed.

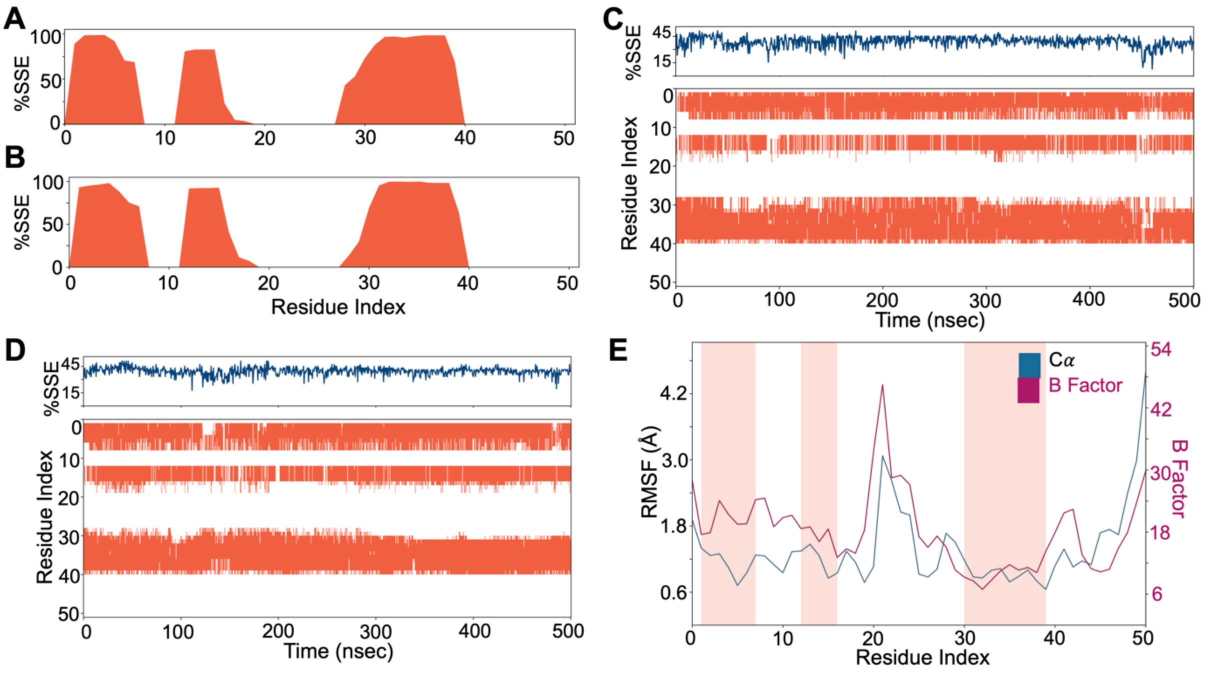

Figure S9. (A) Secondary structure element percentage of insulin versus residue number in the absence of the polymer. (B) Secondary structure element percentage of insulin versus residue number in the presence of the polymer. (C) Secondary structure element percentage and residue number versus MD simulation time in the absence of the polymer. (D) Secondary structure element percentage and residue number versus MD simulation time in the presence of the polymer. The alpha helices are shown in orange. SSE% and residue index are the percentage of secondary structure elements (helices or beta sheets) and residue numbers in insulin molecule, respectively. **(**E) Comparison of theoretical RMSF and experimental B-factor values of insulin.

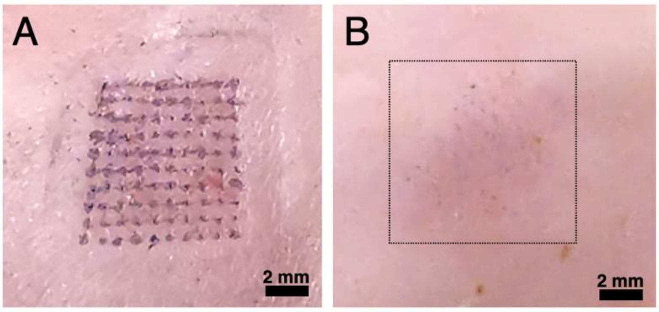

Figure S10. *In vivo* skin penetration of mMN patch. (A) Photograph of trypan blue staining showing mMN patch penetrated the dorsum rat at 15 min post-application. (B) The penetrated rat skin was recovered within 12 hr post-patch application.

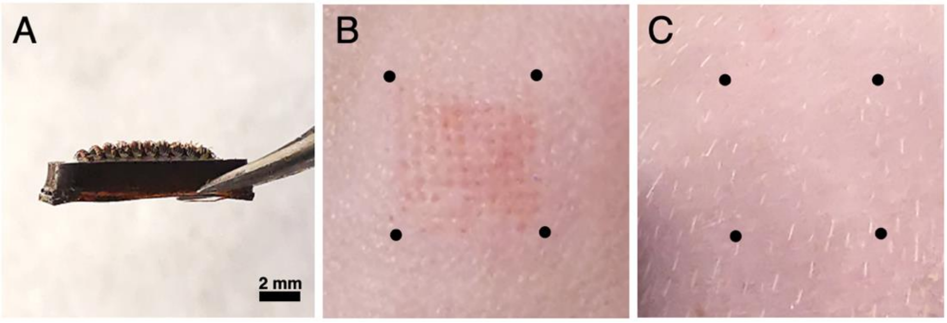

Figure S11. (A) Photograph of mMN array upon removal from rat skin after 8 hr insertion showing that the MNs were removed intact despite extensive swelling and deformation. Photographs of the microchannels on the rat skin (B) immediately and (C) 18 hr post-patch application. Black dots mark the corner areas of the patch application.

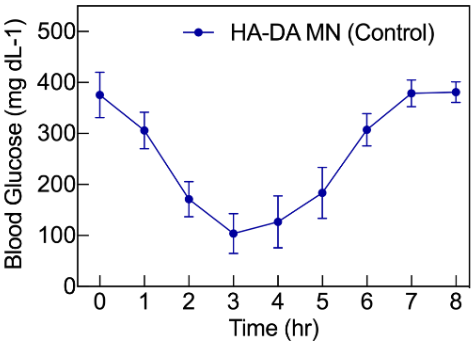

Figure S12. *In vivo* efficacy of crosslinked non-glucose responsive MN patches in T1D rats (n = 3). The MN was demonstrably non-glucose responsive due to the absence of AFBA. Diabetic rats were fasted for 5 hr prior to experiments and treatments occurred at t = 0 hr. The patch administration occurred at t = 0 hr.

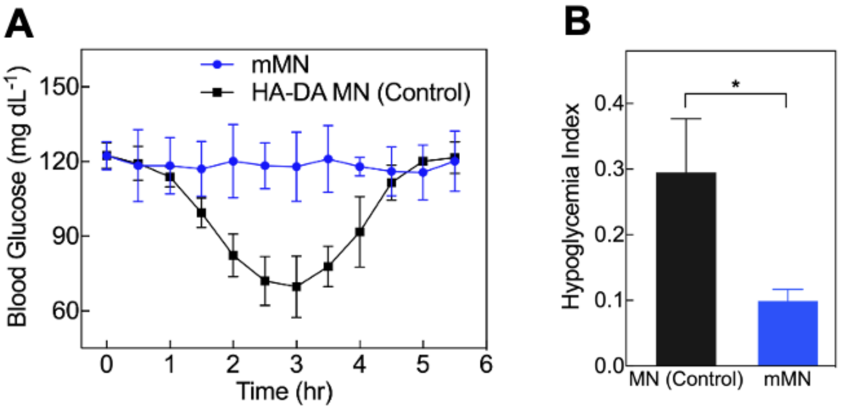

Figure S13. (A) *In vivo* glucose control capability and risk of hypoglycemia in healthy rats treated with glucose-responsive mMN patch or non-glucose-responsive HA-DA MN patch (control) (n = 4). **(B)** Corresponding hypoglycemia index. The quantification was calculated by the difference between the initial blood glucose levels to the nadir in each experiment divided by the time the nadir occurred in that experiment. The administrations occurred at t = 0 hr. Statistical significance between groups was determined using a two-tailed Student's t-test. *p-value < 0.05.

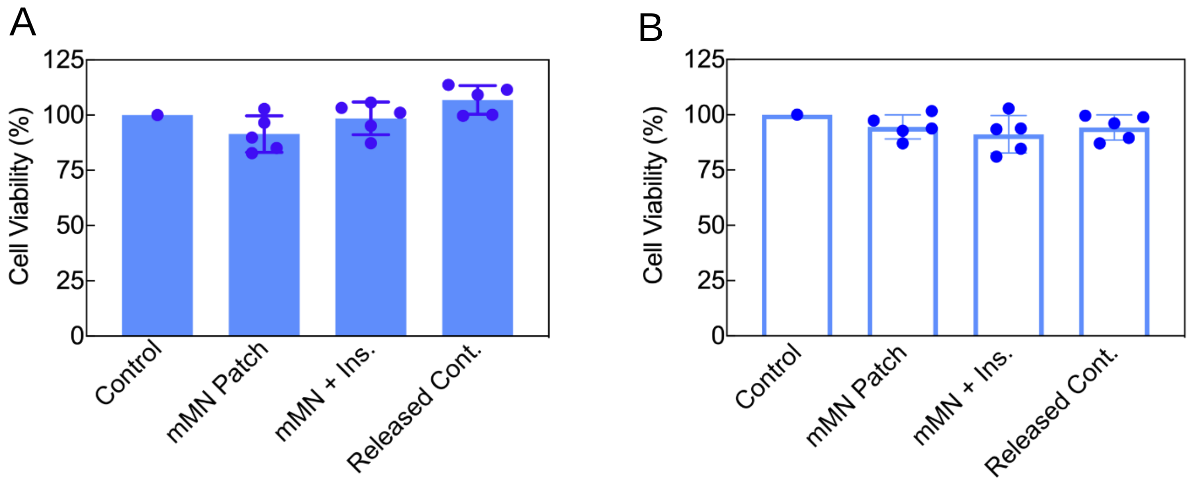

Figure S14. *In vitro* cytotoxicity study of the matrix microneedle patch components on (A) NIH-3T3 fibroblast cells and (B) HaCaT Human keratinocyte cells. mMN: sham patch, mMN + Ins.: sham patch + insulin, released pellet: components released from the insulin-loaded mMN. All data is shown as mean ± standard deviation, n = 5.

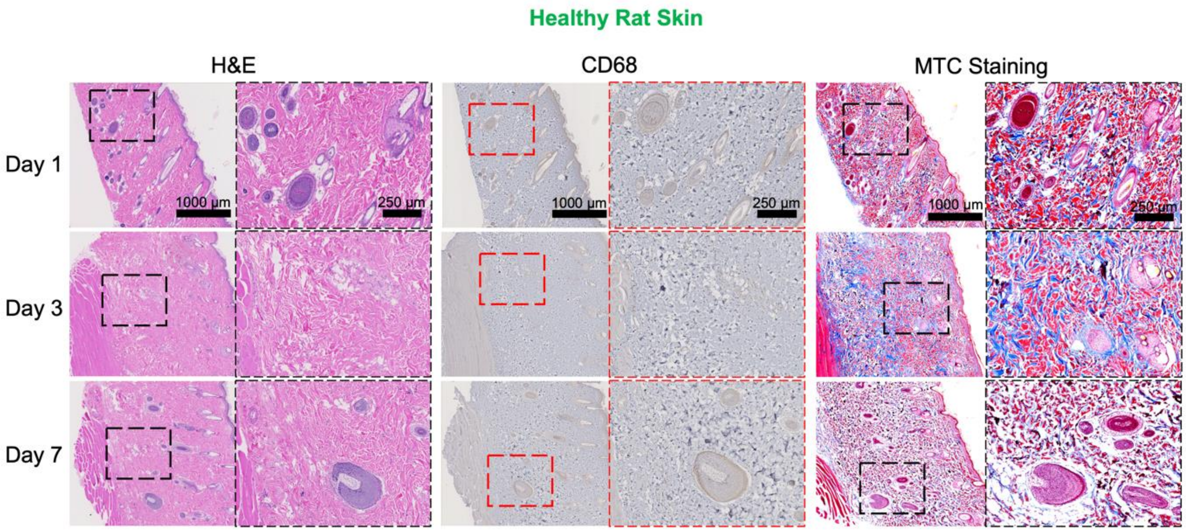

Figure S15. H&E-stained (left), CD68-stained (middle) and MTC (left) staining results. The healthy rat skin tissue was harvested under the same condition as the mMN treatment group on day 1, day 3, and day 7. The healthy rat skin was untreated and used as a control. Inset images with black/red box sections are enlarged views of the box sections on the left.

3. Supplementary Table

Table S1. Evaluation of hydrogel using various ratios (w/w) of HA-DA and HA-AFBA polymers.

Ratio of DA:AFBA	HA-DA (mg)	HA-AFBA (mg)	Gel flowability	Comments
1:1	25	25	Low	The mixture formed gel upon pH adjustment and mixing (< ~10 sec).<br><br>The dried sample partially dissolved after being immersed in PBS buffer for 24 hr, indicating only partial crosslinked gel.
2:1	33.33	16.67	Medium	The mixture formed gel with moderate gelation phase after pH adjustment and complete mixing (~2-3 min).<br><br>Relatively easy handling for sample transferring and MN casting. Good gel integrity.
3:1	40	10	Medium	The mixture formed gel upon pH adjustment and mixing (~2 min).<br><br>Good gel integrity and remained intact when immersed in PBS buffer.
1:2	16.67	33.33	Medium to high	The mixture did not form a complete gel after pH adjustment and mixing.

Table S2. Donors and acceptors in various residues of polymer and insulin molecules involved in H-bond formation between insulin and the polymer identified in MD simulation between insulin and the polymer

**Donor**	**Residue**	**Molecule**	**Acceptor**	**Residue**	**Molecule**	**Distance (Å)**
OH	Glucuronic acid	Polymer	Oxygen	Thr8, chain A	Insulin	2.08
NH_2	**Asn21**, chain A	Insulin	Oxygen	glucuronic acid	Polymer	1.641
OH	N-acetylglucosamine	Polymer	Oxygen	Cys7, chain B	Insulin	1.982
OH	Glucuronic acid	Polymer	Oxygen	Glu13, chain B	Insulin	1.517
OH	Glucuronic acid	Polymer	Oxygen	Glu13, chain B	Insulin	1.674
OH	Glucuronic acid	Polymer	Oxygen	Leu17, chain B	Insulin	2.164
OH	N-acetylglucosamine	Polymer	Oxygen	Leu17, chain B	Insulin	1.915
OH	Glucuronic acid	Polymer	Oxygen	Pro28, chain B	Insulin	1.737
OH	Glucuronic acid	Polymer	Oxygen	Ala30, chain B	Insulin	1.756
NH	Ile2, chain A	Insulin	Oxygen	N-acetylglucosamine	Polymer	1.938
OH	Ser12, chain A	Insulin	Oxygen	N-acetylglucosamine	Polymer	1.817
OH	Tyr19, chain A	Insulin	Oxygen	N-acetylglucosamine	Polymer	1.897
OH	DA, chain B	Polymer	Oxygen	Lys29	Insulin	2.5
OH	DA, chain B	Polymer	Oxygen	Lys29	Insulin	2.135

References

[1] Y. Lee, H. Lee, Y. B. Kim, J. Kim, T. Hyeon, H. Park, P. B. Messersmith, T. G. Park, *Adv. Mater.* **2008**, NA.
[2] E. N. Baker, T. L. Blundell, J. F. Cutfield, E. J. Dodson, G. G. Dodson, D. M. C. Hodgkin, R. E. Hubbard, N. W. Isaacs, C. D. Reynolds, K. Sakabe, *Phil. Trans. R. Soc. Lon. B, Biological Sciences* **1988**, 319, 369.
[3] S. Release, *Maestro-Desmond Interoperability Tools, Schrödinger, New York, NY* **2017**.
[4] J. L. Banks, H. S. Beard, Y. Cao, A. E. Cho, W. Damm, R. Farid, A. K. Felts, T. A. Halgren, D. T. Mainz, J. R. Maple, *J. Comput. Chem.* **2005**, 26, 1752.
[5] A. Y. Toukmaji, J. A. Board Jr, *Comput. Phys.Commun.* **1996**, 95, 73.
[6] A. GhavamiNejad, B. Lu, M. Samarikhalaj, J. F. Liu, S. Mirzaie, S. Pereira, L. Zhou, A. Giacca, X. Y. Wu, *Drug Deliv. Transl. Res.* **2021**.
[7] P. Mark, L. Nilsson, *J. Phys. Chem. A* **2001**, 105, 9954.
[8] G. J. Martyna, M. E. Tuckerman, D. J. Tobias, M. L. Klein, *Mol. Phys.* **1996**, 87, 1117.
[9] G. J. Martyna, M. L. Klein, M. Tuckerman, *J. Chem. Phys.* **1992**, 97, 2635.
[10] E. Lih, S. G. Choi, D. J. Ahn, Y. K. Joung, D. K. Han, *J. Tissue Eng.* **2016**, 7, 2041731416683745.
[11] D. D. Mueller, T. D. Morgan, J. D. Wassenberg, K. J. Kramer, K. J. Kramer, *Bioconjug. Chem.* **1993**, 4, 47.
[12] S. Das, V. L. Alexeev, A. C. Sharma, S. J. Geib, S. A. Asher, *Tetrahedron Lett.* **2003**, 44, 7719.
